# Exploring the association between fat-related traits in chickens and the RGS16 gene: insights from polymorphism and functional validation analysis

**DOI:** 10.3389/fvets.2023.1180797

**Published:** 2023-05-10

**Authors:** Mao Ye, Zhexia Fan, Yuhang Xu, Kang Luan, Lijin Guo, Siyu Zhang, Qingbin Luo

**Affiliations:** ^1^Department of Animal Genetics, Breeding and Reproduction, College of Animal Science, South China Agricultural University, Guangzhou, China; ^2^Guangdong Provincial Key Laboratory of Agro-Animal Genomics and Molecular Breeding, Key Laboratory of Chicken Genetics, Breeding and Reproduction, Ministry of Agriculture, Guangzhou, China

**Keywords:** *RGS16*, chicken, SNP, abdominal fat, association

## Abstract

**Introduction:**

Excessive fat deposition in chickens can lead to reduced feed utilization and meat quality, resulting in significant economic losses for the broiler industry. Therefore, reducing fat deposition has become an important breeding objective in addition to achieving high broiler weight, growth rate, and feed conversion efficiency. In our previous studies, we observed high expression of Regulators of G Protein Signaling 16 Gene (*RGS16*) in high-fat individuals. This led us to speculate that *RGS16* might be involved in the process of fat deposition in chickens.

**Methods:**

Thus, we conducted a polymorphism and functional analysis of the RGS16 gene to investigate its association with fat-related phenotypic traits in chickens. Using a mixed linear model (MLM), this study explored the relationship between RGS16 gene polymorphisms and fat-related traits for the first time. We identified 30 SNPs of *RGS16* in a population of Wens Sanhuang chickens, among which 8 SNPs were significantly associated with fat-related traits, including sebum thickness (ST), abdominal fat weight (AFW), and abdominal fat weight (AFR). Furthermore, our findings demonstrated that AFW, AFR, and ST showed significant associations with at least two or more out of the eight identified SNPs of RGS16. We also validated the role of *RGS16* in ICP-1 cells through various experimental methods, including RT-qPCR, CCK- 8, EdU assays, and oil red O staining.

**Results:**

Our functional validation experiments showed that *RGS16* was highly expressed in the abdominal adipose tissue of high-fat chickens and played a critical role in the regulation of fat deposition by promoting preadipocyte differentiation and inhibiting their proliferation. Taken together, our findings suggest that *RGS16* polymorphisms are associated with fat-related traits in chickens. Moreover, the ectopic expression of *RGS16* could inhibit preadipocyte proliferation but promote preadipocyte differentiation.

**Discussion:**

Based on our current findings, we propose that the RGS16 gene could serve as a powerful genetic marker for marker-assisted breeding of chicken fat-related traits.

## 1. Introduction

Being overweight and obese is a risk factor for major noncommunicable diseases (NCDs), including cardiovascular disease, type 2 diabetes, and cancer (1, 2). China has made many efforts to combat obesity, including implementing national policies and programs to promote healthy life-styles and prevent the development of NCDs, however, these efforts have been insufficient in controlling the rapid increase in overweight and obesity rates in the country (3). As a model animal, chickens are helpful in our study of abdominal fat deposition and may have certain guiding significance for the treatment of obesity-related diseases.

In recent decades, high-density genetic selection has greatly improved the weight, growth rate, and feed conversion efficiency of broilers (4). However, this has also resulted in excessive fat deposition, particularly in the abdomen. This phenomenon can lead to reduced feed utilization efficiency and lower meat quality, thus resulting in wasted resources and environmental pollution, which greatly reduce the desire for consumption and the economic efficiency of producers. Therefore, reducing fat deposition is a key issue to be addressed in broiler production. At the same time, research on fat deposition in chickens has important scientific significance for treating obesity-related diseases, reducing feed waste, and improving economic efficiency (5).

The protein encoded by the Regulators of G Protein Signaling 16 Gene (*RGS16*) belongs to the “regulator of G protein signaling” family. G protein signaling is activated through the binding of extracellular ligands to G protein-coupled receptors (GPCRs) and inhibited inside cells by regulator of G protein signaling (RGS) proteins (6). The GPCR pathway has been shown to influence the metabolism of glucose and fatty acids and the onset of obesity and diabetes (7, 8). RGS proteins are GTPase-activating proteins (GAPs) of alpha subunits that control the intensity and duration of GPCR signaling. The results of studies in recent years have shown that the triglyceride content is significantly higher in the liver of transgenic *RGS16* mice than of nontrans genic mice, indicating that *RGS16* inhibits fatty acid oxidation in the liver (9). It was also found that *RGS16* overexpression promotes lipid droplet formation in rat 832/13 cells and affects the expression of key genes for enzymes that mediate lipid droplet formation (10). Single nucleotide polymorphisms (SNPs) have begun to be used in animal breeding research as highly stable molecular markers that can provide a wealth of information (11, 12).

To date, *RGS16* has been relatively little studied in chicken compared with the mammalian counterpart. In our previous work, to explore the differences in fat deposition by high and low-fat broilers (13), we sequenced the transcriptomes of the abdominal fat of Wens Sanhuang chickens and found that the expression of *RGS16* in abdominal fat was higher in high-fat individuals. Therefore, we hypothesized that *RGS16* might be involved in the process of fat deposition in chicken. We provide some theoretical basis and direction for the selection of low-fat broilers by verifying the role of *RGS16* in ICP-1 cell and analyzing the relationship between polymorphisms and fat-related traits including abdominal fat weight, abdominal fat rate and sebum thickness.

## 2. Materials and methods

### 2.1. Experimental animals and fat-related traits data

The F_2_ population of 100-day-old Sanhuang chickens (*n* = 439) used for the slaughter experiments in this study were reared under the same environmental and management conditions by Wens Food Group Co., Ltd. (Yunfu, China). The animal experiments in this study were approved by the Animal Care Committee of South China Agricultural University (permit number: SCAU#2017015, 13 September 2017) (14). The fat-related traits including AFW, AFR and ST were measured and calculated according to the Performance terminology and measurements for poultry (NY/T823-2020).

### 2.2. DNA extraction, PCR, and DNA sequencing

Blood samples were collected from veins under the wings of all Sanhuang chickens (*n* = 439), and DNA extraction was performed according to the instructions included in the DNA extraction kit (OMEGA, Georgia, United States). All DNA samples were used to amplify the dsDNA of the *RGS16* 5′UTR, 3′UTR and CDS with 2× Taq MasterMix (CWBIO, Nanjing, China). The PCR reaction conditions were as follows: pre-denaturation at 94°C for 2 min, amplification at 94°C for 30 s, 60°C for 30 s, and 72°C for 1 min, repeated for 30 cycles, final amplification at 72°C for 2 min, and hold at 4°C indefinitely. Finally, the PCR products were purified and sequenced by Sangon Biotech (Shanghai, China).

### 2.3. Cell culture, treatment, and transfection

The immortalized chicken preadipocyte 1 (ICP-1) cells used in this study were provided by Li Hui’s research group from Northeast Agricultural University (Heilongjiang, China). The basal medium used was DMEM/F12 (Gibco, United States) supplemented with 15% fetal bovine serum (FBS) (Gibco, United States) and 1% streptomycin/penicillin solution (Gibco, United States), the cells were cultured at 37°C and 5% CO_2_ in a calculator (15).

### 2.4. Overexpression plasmid construction and siRNA synthesis

To construct a chicken *RGS16* overexpression plasmid, the complete CDS region of *RGS16* was cloned into the EcoRI and BamHI sites of pcDNA3.1 (Promega, New York, United States), and the plasmids were extracted according to the instructions included with the HiPure Plasmid/BAC EF Mini Kit (Magen, Guangzhou, China). The knockdown of *RGS16* (5′-GGACCATTGATGGCCATAA-3′) was performed using specific siRNA designed and synthesized by RiboBio Co., Ltd. (Guangzhou, China).

### 2.5. RNA extraction, cDNA synthesis, and quantitative real-time PCR

Total RNA was extracted from tissues or cells using Magzol Reagent (Magen, Guangzhou, China) following the manufacturer’s instructions. PrimeScript RT Reagent Kit with gDNA Eraser (TaKaRa, Otsu, Japan) was used for cDNA preparation from total RNA. RT-qPCR was performed using a ChamQ Universal SYBR qPCR Master Mix (Vazyme, Nanjing, China) in a Bio-Rad CFX96 Real-Time Detection System. Data were analyzed using the 2^−△△Ct^ method with chicken *GAPDH* as the reference gene (16). The RT-qPCR primers were designed in Primer-BLAST^1^ and their detail were listed in Supplementary Table 1.

### 2.6. Oil red O staining

For oil red O staining, the ICP-1 cells were washed with PBS (Gbico, New York, United States) and then fixed with 4% paraformaldehyde solution (Sangon Biotech, Shanghai, China) for 30 min at room temperature. After fixation, the cells were rinsed twice with PBS, and oil red O staining solution (Sangon Biotech, Shanghai, China) was added followed by the incubation of cells for 60 min at room temperature. Then, the staining solution was removed, and the cells were washed three times with PBS. Image analysis was carried out using a DMi8 microscope (Leica, Wetzlar, Germany). The oil red O dye in stained cells was extracted with isopropanol solution and the absorption value was measured at 510 nm using a microreader (Bio-Tek, Vermont, United States).

### 2.7. 5-ethynyl-2′-deoxyuridine assay

EdU staining was performed using a Cell-Light EdU Apollo 488 *In Vitro* Kit (RiboBio, Guangzhou, China). For the EdU assay, ICP-1 cells were treated with EdU medium (1:1,000; RiboBio, Guangzhou, China) for 2 h at 37°C and then fixed in 4% paraformaldehyde solution (Yike, Guangzhou, China) for 30 min. The cells were subsequently permeabilized using 0.1% Triton X-100 solution (Gbico, New York, United States). The cells were incubated with the staining solution for 30 min at room temperature in the dark. Finally, the stained cells were scraped off with a cell scraper, collected in a 1.5 mL centrifuge tube and resuspended in 1 mL PBS. A BD Accouri C6 flow cytometer (BD Biosciences, San Jose, CA, United States) was utilized for the analysis of stained cells (16).

### 2.8. Cell counting kit-8 assay

For the CCK-8 assay, ICP-1 cells were inoculated in 96-well plates and transfected with plasmids or siRNA. Cell proliferation and viability were then monitored at 12, 24, 36, and 48 h using the CCK-8 kit (TransGen Biotech, Beijing, China) following the manufacturer’s instructions. This analysis was performed using a microreader (Bio-Rad, Hercules, CA, United States) to measure the absorption value at 450 nm.

### 2.9. Statistical analysis

The MLM package in IBM SPSS software (version 26, IBM: International Business Machines Corporation) was used to analyze the association of gene polymorphisms and haplotypes with chicken fat-related traits. All results were represented as mean ± SEM. The MLM model is as follows:


Y=μ+G+S+e

Where Y represents the observed value, μ represents the mean, G represents the fixed effect of genotypes, S represents the fixed effect of sex, and e is the random error. In addition, we used Haploview v.4.2 software for linkage disequilibrium analysis (17, 18).

Statistical analysis and plots generation were conducted using GraphPad Prism v.9.4 (CA, United States) and R studio v.4.2.1 software (MA, United States). Correlation coefficients were calculated using Pearson correlation analysis. An unpaired *t*-test (two-tailed) was used to assess the statistical significance between two groups. In multiple comparisons, significant differences were assessed using the LDS method.

## 3. Results

### 3.1. Phenotypic data

The descriptive statistics of fat-related traits in the F_2_ population of Wens Sanhuang chickens (*n* = 439) was listed in Table 1. Among of them, average AFW was 98.43 g, the max value was 193.00 while the min value was 28.40 indicating a particularly evident inter-individual differences and a wide range of variation, with a coefficient of variation exceeding 30%. Average AFR was 7.72%, with a min value of 2.79% and a max value of 13.68%, and a CV value of 22.8%. Its CV value was much lower than that of AFW, indicating a potential positive correlation between AFW and body weight. For ST, the average value was 7.53 mm, with a min value of 1.16 mm and a max value of 12.83 mm. The CV of ST was 21.87%, which was closed to that of AFR. In general, the CV of fat-related traits in this population was large, which could be used to validate the association analysis between nucleotide polymorphism and fat traits.

**Table 1:** Summary statistics of fat-related traits in the Wens Sanhuang chicken population.

Trait	N	Mean	SD	Min.	Max.	Var	C.V (%)
AFW (g)	439	98.43	29.57	28.40	193.00	874.13	30.04
AFR (%)	439	7.72	1.76	2.79	13.68	0.03	22.80
ST (mm)	439	7.53	1.64	1.16	12.83	2.70	21.78

N, number; SD, standard deviation; CV, coefficient of variation; AFW, abdominal fat weight; AFR, abdominal fat rate; ST, sebum thickness.

### 3.2. SNPs discovery and genotypes

**Figure 1:**
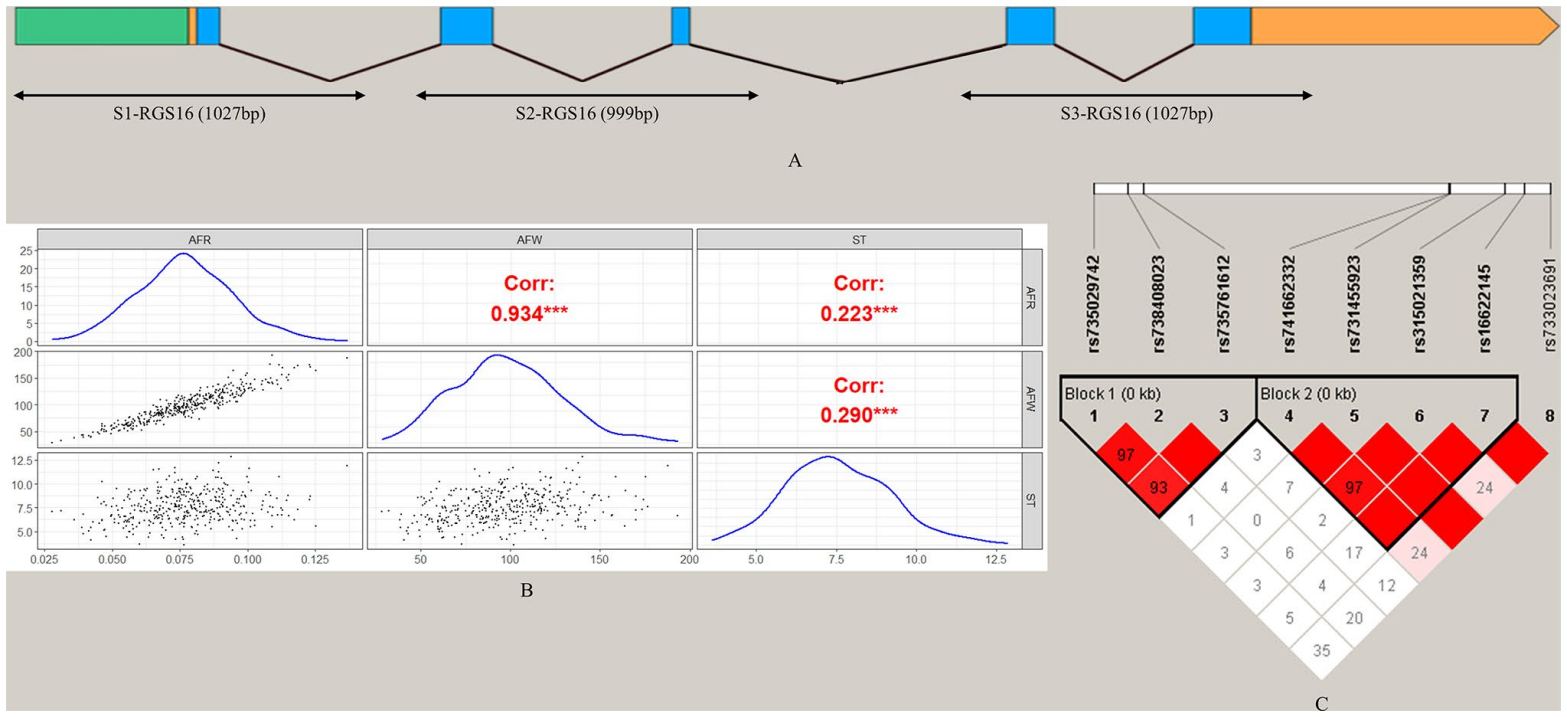
Analysis of 30 SNPs in the *RGS16* in Wens Sanhuang chickens. **(A)** The locations of the three primers used for SNP screening in the *RGS16*. **(B)** Pearson correlation coefficients between fat-related traits in Wens Sanhuang chickens. **(C)** The paired linkage disequilibrium (LD) values (D′) of the SNPs are represented by the values in the boxes. When D′ = 1, the values are not displayed. The intensity of the red color in the box represents the strength of LD, with darker shades indicating stronger LD. The Haploview software was used to define haplotype blocks with the default settings. * Denotes statistical significance with * < 0.05; ** < 0.01; *** < 0.001; **** < 0.0001.

To screen for polymorphisms, the flanking and exon regions of *RGS16*, with a length of 3,053 bp, were, respectively, amplified (Figure 1A). A total of 30 SNPs were identified in this region from the F_2_ population of Wens Sanhuang chickens, and these were mapped to the genome sequence (Version: GRCg6a) in the Ensembl Database for identification (Table 2). The sequencing files of the 439 samples were analyzed using SnapGene v4.1.8 software to identify their genotypes for every individuals. Among of the 30 SNPs, the 6 SNPs were located in exon region but they were all synonymous variant. The statistics of genotype frequencies and allele frequencies are shown in Table 3. The 30 identified SNPs could all be divided into three genotypes. A chi-square test confirmed that all identified SNPs were in accordance with Hardy–Weinberg equilibrium (*p* > 0.05). Pearson correlation coefficients were used to analyze the correlations between fat-related traits, including AFW, AFR, and ST. As shown in Figure 1B, AFW was significantly and positively correlated with AFR (*ρ* = 0.934, *p* < 0.001), while ST is significantly and positively correlated with AFW and AFR (*ρ* = 0.290 for AFW, *ρ* = 0.234 for AFR, *p* < 0.001).

**Table 2:** Details of SNPs.

**SNPs**	**Chr:bp**	**Alleles**	**Class**	**Conseq. type**
rs736356137	8:5981044	T/C	SNP	Upstream gene variant
rs740542560	8:5981078	A/G	SNP	Upstream gene variant
rs731768083	8:5981083	G/C	SNP	Upstream gene variant
rs737985212	8:5981113	C/T	SNP	Upstream gene variant
rs740327014	8:5981388	G/A	SNP	Upstream gene variant
rs741317696	8:5981575	C/T	SNP	5 prime UTR variant
rs731255195	8:5981677	G/A	SNP	Intron variant
rs738816885	8:5981722	G/A	SNP	Intron variant
rs739467979	8:5981728	T/C	SNP	Intron variant
rs731997801	8:5981743	A/G	SNP	Intron variant
rs741182381	8:5981751	T/C	SNP	Intron variant
rs735029742	8:5983002	T/C	SNP	Intron variant
rs738408023	8:5983141	T/C	SNP	Intron variant
rs735761612	8:5983206	C/T	SNP	Intron variant
rs735218667	8:5983393	G/A	SNP	Intron variant
rs317340874	8:5983420	G/C	SNP	Intron variant
rs740889485	8:5983563	T/C	SNP	Intron variant
rs741662332	8:5984436	T/C	SNP	Synonymous variant
rs731455923	8:5984445	T/C	SNP	Synonymous variant
rs741115314	8:5984535	C/T	SNP	Synonymous variant
rs734517162	8:5984595	G/A	SNP	Intron variant
rs315021359	8:5984665	T/C	SNP	Intron variant
rs16622145	8:5984743	A/G	SNP	Intron variant
rs16622146	8:5984747	C/T	SNP	Intron variant
rs16622147	8:5984756	C/T	SNP	Intron variant
rs16622148	8:5984798	T/C	SNP	Intron variant
rs733023691	8:5984846	T/C	SNP	Intron variant
rs312359940	8:5984919	A/T	SNP	Intron variant
rs734891921	8:5984945	C/T	SNP	Intron variant
rs80763227	8:5985125	C/T	SNP	Synonymous variant

**Table 3:** Genotypes and allele frequencies and diversity parameters of SNPs in the *RGS16*.

SNP	Genotype frequencies (n)			Allelic frequencies		*p*-value	Genetic polymorphism			
	AA	BB	AB	A	B		*PIC*	*Ho*	*Ne*	*He*
rs736356137	65 (0.159)	154 (0.377)	190 (0.465)	0.391	0.609	0.882	0.363	0.476	1.910	0.465
rs740542560	284 (0.694)	16 (0.039)	109 (0.267)	0.828	0.172	0.411	0.245	0.285	1.399	0.267
rs731768083	284 (0.694)	16 (0.039)	109 (0.267)	0.828	0.172	0.411	0.245	0.285	1.399	0.267
rs737985212	7 (0.017)	291 (0.711)	111 (0.271)	0.153	0.847	0.622	0.225	0.259	1.349	0.271
rs740327014	329 (0.804)	6 (0.015)	74 (0.181)	0.895	0.105	0.739	0.170	0.188	1.232	0.181
rs741317696	7 (0.017)	273 (0.667)	129 (0.315)	0.175	0.825	0.169	0.247	0.289	1.406	0.315
rs731255195	254 (0.621)	10 (0.024)	145 (0.355)	0.798	0.202	0.125	0.270	0.322	1.475	0.355
rs738816885	257 (0.628)	10 (0.024)	142 (0.347)	0.802	0.198	0.171	0.267	0.318	1.466	0.347
rs739467979	10 (0.024)	257 (0.628)	142 (0.347)	0.198	0.802	0.171	0.267	0.318	1.466	0.347
rs731997801	255 (0.623)	11 (0.027)	143 (0.350)	0.798	0.202	0.223	0.270	0.322	1.475	0.350
rs741182381	10 (0.024)	256 (0.626)	143 (0.350)	0.199	0.801	0.154	0.268	0.319	1.469	0.350
rs735029742	4 (0.010)	323 (0.788)	83 (0.202)	0.111	0.889	0.871	0.178	0.197	1.246	0.202
rs738408023	311 (0.759)	5 (0.012)	94 (0.229)	0.873	0.127	0.776	0.197	0.221	1.285	0.229
rs735761612	49 (0.120)	166 (0.405)	195 (0.476)	0.357	0.643	0.772	0.354	0.459	1.849	0.476
rs735218667	10 (0.024)	254 (0.620)	146 (0.356)	0.202	0.798	0.115	0.271	0.323	1.477	0.356
rs317340874	254 (0.62)	14 (0.034)	142 (0.346)	0.793	0.207	0.553	0.275	0.329	1.490	0.346
rs740889485	4 (0.010)	304 (0.766)	89 (0.224)	0.122	0.878	0.666	0.191	0.214	1.273	0.224
rs741662332	265 (0.604)	20 (0.046)	154 (0.351)	0.779	0.221	0.924	0.285	0.344	1.525	0.351
rs731455923	28 (0.064)	249 (0.567)	162 (0.369)	0.248	0.752	0.972	0.304	0.373	1.596	0.369
rs741115314	11 (0.025)	324 (0.738)	104 (0.237)	0.144	0.856	0.749	0.216	0.246	1.326	0.237
rs734517162	11 (0.025)	282 (0.642)	146 (0.333)	0.191	0.809	0.294	0.262	0.309	1.448	0.333
rs315021359	262 (0.597)	19 (0.043)	158 (0.360)	0.777	0.223	0.731	0.287	0.347	1.531	0.360
rs16622145	287 (0.654)	12 (0.027)	140 (0.319)	0.813	0.187	0.581	0.258	0.304	1.436	0.319
rs16622146	16 (0.036)	287 (0.654)	136 (0.310)	0.191	0.809	1.000	0.262	0.309	1.448	0.310
rs16622147	118 (0.269)	107 (0.244)	214 (0.487)	0.513	0.487	0.877	0.375	0.500	1.999	0.487
rs16622148	288 (0.656)	15 (0.034)	136 (0.310)	0.811	0.189	0.977	0.260	0.307	1.442	0.310
rs733023691	239 (0.544)	22 (0.050)	178 (0.405)	0.747	0.253	0.309	0.306	0.378	1.607	0.405
rs312359940	206 (0.469)	45 (0.103)	188 (0.428)	0.683	0.317	0.977	0.339	0.433	1.763	0.428
rs734891921	43 (0.098)	173 (0.394)	223 (0.508)	0.352	0.648	0.059	0.352	0.456	1.839	0.508
rs80763227	101 (0.230)	126 (0.287)	212 (0.483)	0.472	0.528	0.810	0.374	0.498	1.994	0.483

SNP, single nucleotide polymorphism (numbers in parentheses indicate the number of individuals); PIC, polymorphism information content; Ho, observed heterozygosity; He, expected heterozygosity; Ne, effective number of alleles. The test for Hardy–Weinberg equilibrium with *p*-value > 0.05, indicating that the population is in Hardy–Weinberg equilibrium.

### 3.3. Association between SNPs in *RGS16* and chicken fat-related traits

To perform association analysis of genotypes with fat-related traits in chickens, the MLM package in IBM SPSS software was utilized. The full results of this analysis were listed in Supplementary Table 2. The SNPs significantly associated with one or more fat-related traits were shown in Table 4. In terms of ST, the CC and TC genotypes of both rs735029742 and rs731455923 were found to be significantly higher than the TT genotype (*p* < 0.05). Additionally, the TT genotype of rs738408023 was significantly higher than the TC genotype (*p* < 0.05). For AFW and AFR, the TT and TC genotypes were significantly higher than CC genotypes for rs741662332 and rs315021359, respectively, while the CC genotypes were significantly higher than TT and TC genotypes for rs733023691 (*p* < 0.05). The CC and TC genotypes of rs735761612 were found to have higher AFR than the TT genotype (*p* < 0.05), and the AA and AG genotypes of rs16622145 were found to have higher AFR than the GG genotype (*p* < 0.05).

**Table 4:** Association of eight SNPs in *RGS16* with fat-related traits in Wens Sanhuang chicken.

SNP	Trait	Mean ± SEM			*p*-value
rs735029742		CC (322)	TC (83)	TT (5)	
	ST (mm)	7.63 ± 1.60^A^	7.24 ± 1.68^B^	6.42 ± 0.85^AB^	0.043
rs738408023		CC (6)	TC (93)	TT (311)	
	ST (mm)	7.00 ± 1.64	7.18 ± 1.64^A^	7.65 ± 1.60^A^	0.034
rs735761612		CC (166)	TC (196)	TT (48)	
	AFR (%)	7.74 ± 1.83^A^	7.74 ± 1.70^B^	7.04 ± 1.53^AB^	0.034
rs741662332		CC (20)	TC (154)	TT (265)	
	AFW (g)	83.1 ± 29.92^AB^	100.31 ± 29.77^A^	98.49 ± 29.2^B^	0.049
	AFR (%)	6.80 ± 1.9^AB^	7.88 ± 1.75^A^	7.69 ± 1.74^B^	0.034
rs731455923		CC (249)	TC (162)	TT (28)	
	ST (mm)	7.63 ± 1.61^A^	7.49 ± 1.65^B^	6.83 ± 1.76^AB^	0.047
rs315021359		CC (19)	TC (158)	TT (262)	
	AFW (g)	83.25 ± 30.98^AB^	99.95 ± 29.66^A^	98.61 ± 29.21^B^	0.043
	AFR (%)	6.84 ± 2.05^AB^	7.85 ± 1.74^A^	7.70 ± 1.74^B^	0.045
rs16622145		AA (287)	AG (140)	GG (12)	
	AFR (%)	7.80 ± 1.80^A^	7.66 ± 1.65^B^	6.48 ± 1.71^AB^	0.036
rs733023691		CC (22)	TC (178)	TT (239)	
	AFW (g)	114.96 ± 33.61^AB^	96.65 ± 29.6^B^	98.23 ± 28.8^A^	0.023
	AFR (%)	8.60 ± 1.90^AB^	7.60 ± 1.78^B^	7.72 ± 1.72^A^	0.042

The same letters in the same row indicate a significant difference (*p* < 0.05), whereas different or no letters in the same row indicate no significant difference (*p* > 0.05).

### 3.4. Linkage disequilibrium and haplotype analysis of the *RGS16*

To better understand the relationship between SNPs and fat-related traits, the eight SNPs significantly associated with fat-related traits were further analyzed for linkage disequilibrium (LD) using Haploview software (Figure 1C). The LD plot showed two haplotype blocks, with block 1 including rs735029742, rs738408023, and rs735761612, and block 2 including rs741662332, rs731455923, rs315021359, and rs16622145. All SNP pairs within each block had high LD values ranging from 0.93 to 1.00. Both blocks were analyzed using the MLM model in relation to three fat-related traits. Supplementary Table 3 showed that block 1 was not significantly associated with any traits, while block 2 was significantly associated with AFR and ST. Table 5 presented the traits and relevant data indicating a significant association with block 2 haplotypes. For AFR, both H3H3 and H4H4 genotypes were significantly lower than H1H1, H1H2, H1H3, H1H4, H2H3, and H4H3 (*p* < 0.05). As for ST, both H2H2 and H3H3 genotypes were significantly lower than H1H1, H1H4, and H4H2 (*p* < 0.05). In addition, the H2H2 genotype was significantly lower than H1H3 (*p* < 0.05). These associations demonstrated that *RGS16* may have a potential role in regulating chicken fat deposition.

**Table 5:** Association analysis of blocks with fat-related traits in Wens Sanhuang chicken.

LD block	SNP	Haplotype	Diplotype (n)	Fat-related trait	
				AFR (%)	ST (mm)
Block 2			H1H1 (52)	7.85 ± 1.59^D^	7.91 ± 1.89^B^
			H1H2 (76)	7.98 ± 1.89^B^	7.35 ± 1.72
			H1H3 (59)	8.04 ± 1.66^A^	7.59 ± 1.68^D^
	rs741662332	H1: TCTA (0.339)	H1H4 (56)	7.74 ± 1.65^E^	7.94 ± 1.59^A^
	rs731455923	H2: TTTA (0.247)	H2H2 (28)	7.63 ± 1.72	6.83 ± 1.76^ABCD^
	rs315021359	H3: CCCA (0.216)	H2H3 (48)	7.68 ± 1.94^F^	7.50 ± 1.68
	rs16622145	H4: TCTG (0.186)	H3H3 (17)	6.66 ± 1.90^ABCDEF^	6.80 ± 0.95^ABC^
			H4H2 (37)	7.35 ± 1.69	7.77 ± 1.46^C^
			H4H3 (44)	7.87 ± 1.63^C^	7.40 ± 1.34
			H4H4 (12)	6.48 ± 1.71^ABCDEF^	7.37 ± 1.28
			*p*-value	0.0317	0.0496

The same letters on in the same column indicate a significant difference (*p* < 0.05), whereas different or no letters in the same column indicate no significant difference (*p* > 0.05).

### 3.5. *RGS16* inhibits preadipocyte proliferation

**Figure 2:**
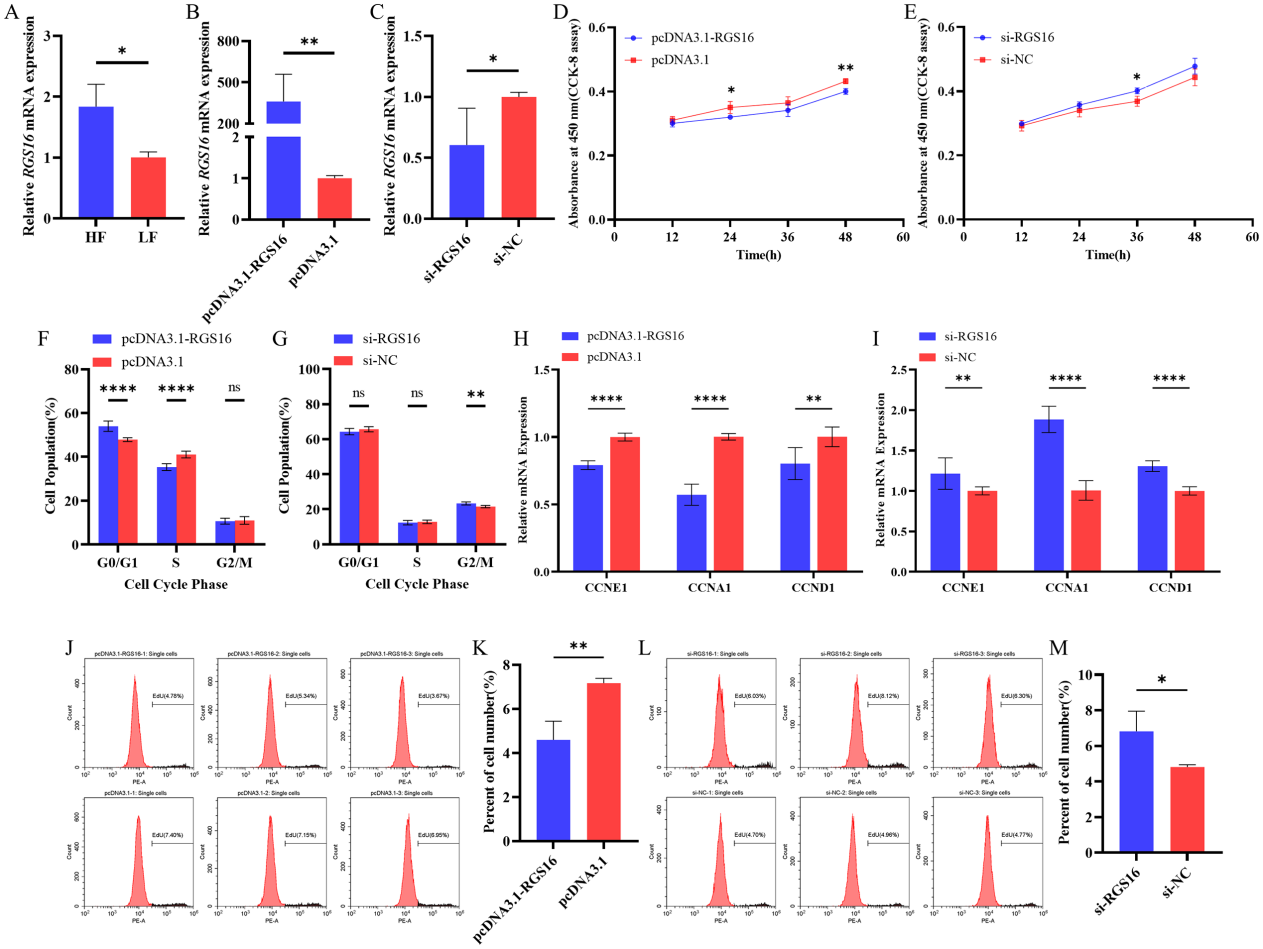
Regulation of preadipocyte proliferation by *RGS16.* **(A)** Differential expression of *RGS16* mRNA in the abdominal fat of high-fat and low-fat chickens measured by RT-qPCR (*n* = 6 per group). **(B,C)** *RGS16* mRNA expression levels in ICP-1 cells overexpressing or with knockdown of *RGS16* (*n* = 6 per group). **(D,E)** CCK-8 assay to assess the effect of *RGS16* overexpression or knockdown on ICP-1 cell viability (*n* = 4 per group). **(F,G)** Cell cycle analysis of ICP-1 cells 48 h after *RGS16* overexpression or knockdown (*n* = 6 per group). **(H,I)** Expression of cell cycle genes in ICP-1 cells with *RGS16* overexpression and knockdown detected by RT-qPCR (*n* = 6 per group). **(J–M)** ICP-1 cell cycle analysis using the flow cytometry EdU assay 48 h after transfection with the *RGS16* overexpression vector or siRNA (*n* = 3 per group). * Denotes statistical significance with * < 0.05; ** < 0.01; *** < 0.001; **** < 0.0001.

In our previous study, we found that *RGS16* was highly expressed in individuals with high level of abdominal fat (accession ID: PRJNA656618). Here, the *RGS16* mRNA level difference between high- and low-fat groups was verified using RT-qPCR (Figure 2A). To investigate the function of *RGS16* in preadipocytes, ICP-1 cells were transfected with the *RGS16* overexpression plasmid and siRNA. The RT-qPCR results showed that the expression of *RGS16* mRNA could be upregulated by more than 400 times and knocked down by about 40%, respectively (Figures 2B,C). CCK-8 assay was performed to measure the proliferation viability of ICP-1 cells after 12-, 24-, 36-, and 48-h transfection. From these data, it is evident that *RGS16* overexpression significantly inhibits cell proliferation at 24 and 48 h, whereas *RGS16* knockdown significantly promotes cell proliferation at 36 h (Figures 2D,E). To further assess the function of *RGS16*, the cell cycle phase was detected using flow cytometry after 48-h transfection. The results showed that the overexpression of *RGS16* prolongs the G1 phase, preventing cells from entering the S phase and inhibiting their proliferation (Figures 2F,G). Furthermore, we also detected the mRNA levels of several cell-cycle-associated genes, *CCNE1*, *CCNA1*, and *CCND1* using RT-qPCR and found that the overexpression of *RGS16* significantly inhibits the expression of *CCNA1*, *CCNE1*, and *CCND1*, while *RGS16* knockdown had the opposite effect, revealing its inhibitory effect on cell proliferation (Figures 2H,I). We performed an EdU assay to verify this conclusion. Forty-eight h after transfection, EdU staining was detected using flow cytometry, and the results showed that the proportion of EdU-stained cells significantly decreases with *RGS16* overexpression and increases with *RGS16* knockdown (Figures 2J–M). All of the above experimental results indicated that *RGS16* is able to inhibit preadipocyte proliferation.

### 3.6. *RGS16* promotes preadipocyte differentiation

**Figure 3:**
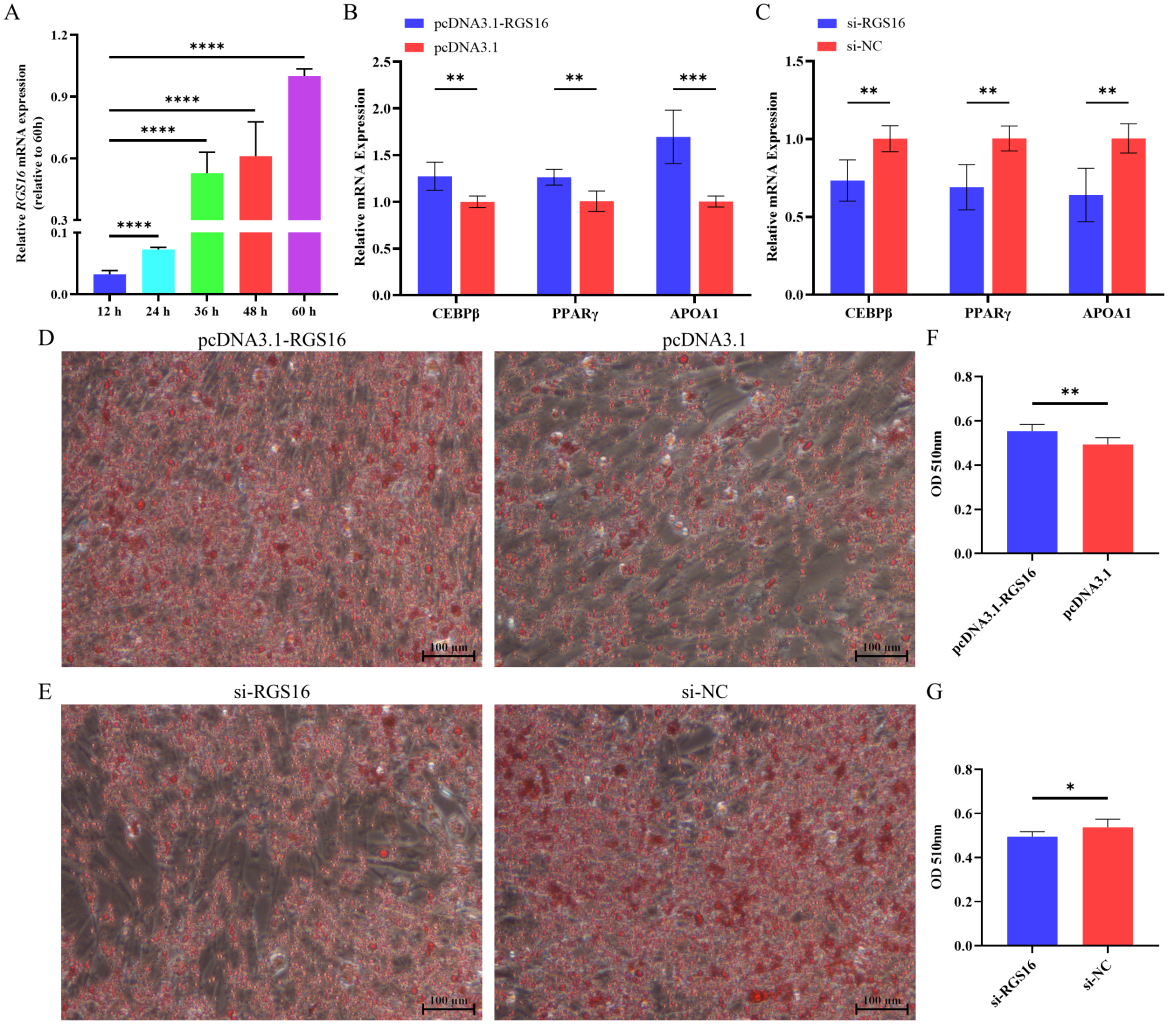
Regulation of preadipocyte differentiation by *RGS16*. **(A)** Expression of *RGS16* mRNA at different time points during differentiation measured by RT-qPCR (*n* = 6 per group). **(B,C)** RT-qPCR analysis of preadipocyte differentiation-related gene expression in ICP-1 cells overexpressing or with knockdown of *RGS16* (*n* = 6 per group). **(D–G)** Oil red O staining and quantification of cells on day 2 after transfection (*n* = 6 per group). * Denotes statistical significance with * < 0.05; ** < 0.01; *** < 0.001; **** < 0.0001.

In addition, we hypothesized that *RGS16* may be involved in regulating the process of chicken preadipocyte differentiation, based on its mRNA level difference between high- and low-fat individuals. ICP-1 differentiation was induced with 80 μM sodium oleate, and cells were collected at five different time points (12, 24, 36, 48, and 60 h) for RNA extraction. Interestingly, we found that *RGS16* expression was significantly increased during ICP-1 cell differentiation (Figure 3A). The cells were collected for RNA extraction after 48-h *RGS16* overexpression and *RGS16* knock down, and RT-qPCR was performed to detect the expression level of genes associated with preadipocyte differentiation (including *PPARγ*, *CEBPβ*, and *APOA1*). The RT-qPCR results showed that *RGS16* overexpression increases the expression of preadipocyte differentiation-related genes, whereas *RGS16* knockdown decreases their expression (Figures 3B,C). After 12 h of transfection, the cells were induced in 80 μM sodium oleate medium for 48 h and stained with oil red O. The results show that *RGS16* overexpression promotes lipid droplet formation, whereas *RGS16* knockdown inhibits lipid droplet formation (Figures 3D–G). Our results demonstrated that *RGS16* indeed is capable of driving preadipocyte differentiation and lipid droplet formation.

## 4. Discussion

In this study, we conducted an association analysis between *RGS16* polymorphisms and fat-related traits, and identified 8 SNPs that are significantly associated with fat-related traits. In addition, we also have concluded that *RGS16* plays an important role in regulating abdominal fat deposition with the mechanism by promoting the differentiation of ICP-1 cells.

With the continuous advancement of technology, molecular marker-assisted breeding has become one of the technologies that has attracted attention and application in the field of poultry production. This technology utilizes molecular marker techniques and principles of genetics to quickly and accurately screen poultry breeds with excellent genetic characteristics, avoiding the tedious breeding process and long feeding cycles involved in traditional breeding methods (11, 19). In this study, according to the Pearson correlation coefficient, we found a significant correlation between ST and AFW and AFR. This suggests that ST may be related to fat deposition, which is consistent with previous research findings (20). We conducted an analysis of the associations between *RGS16* gene polymorphisms and fat-related traits. A total of 30 SNPs were identified, out of which eight SNPs (rs735029742, rs738408023, rs735761612, rs741662332, rs731455923, rs315021359, rs16622145, and rs733023691) were found to be significantly associated with fat-related traits, such as AFW, AFR, and ST. Through association analysis, it was found that individuals carrying the *RGS16* with the TT genotype of rs735029742, the CC genotype of rs738408023, and the TT genotype of rs731455923 exhibited lower ST. Individuals with the TT genotype of rs735761612 and the GG genotype of rs16622145 showed lower AFR. Individuals with the CC genotype of rs741662332, the CC genotype of rs3152021359, and the TC genotype of rs733023691 showed lower AFR and AFW. With broiler breeders now focusing on selecting individuals with low fat weight or low rates of fat, these SNP genotypes could serve as molecular markers for improving fat-related traits in the Wens Sanhuang Chicken.

Synonymous mutations are associated with specific diseases or traits in multiple cases. When a cluster of infrequently used codons shifts from frequent codons to rare ones, it can affect the timing of co-translational folding and lead to changes in protein function (21–23). In this study, we identified four SNPs in the coding region of the *RGS16* gene, namely rs741662332, rs731455923, rs741115314, and rs80763227. However, these sites do not alter the amino acid sequence, which are also known as synonymous mutations. Although we could not directly observe the effects of synonymous mutations, we could infer that they may have an impact on these traits from the analysis of the effects of different genotypes of the synonymous SNP rs731455923 and rs315021359 on abdominal fat-related traits. In addition, we found that 6 SNPs in the intronic region of the RGS16 gene are significantly associated with adiposity-related traits. It should be noted that compared to SNPs in the coding region, the functional effects of SNPs in the intronic region were often more complex and difficult to predict. Specifically, intronic SNPs may affect the structure and function of RNA molecules, such as affecting splice site selection, regulating splicing efficiency, and influencing RNA stability and translation efficiency, which in turn affect protein expression and function (24–26). Haplotypes can often provide more information than a single SNP, as the phenotype of animals can be affected by multiple mutations (27). Subsequently, we used Haploview software to analyze the above eight SNPs for link-age disequilibrium. The finding that block 2 (rs741662332, rs731455923, rs315021359, and rs16622145) is related with AFR and ST offers strong evidence supporting the use of these SNPs as markers in breeding.

Adipogenesis and lipogenesis are regulated by a complex network of transcription factors that function at different stages of differentiation (28, 29). Peroxisome proliferator-activated receptor γ (*PPARγ*) and members of the CCAAT/enhancer-binding protein (*C/EBP*) family are key regulators of this process (13, 30, 31). As one of the three subtypes of the PPAR subfamily, *PPARγ* regulates the lipogenesis of all adipocytes and binds to thousands of loci during the differentiation of white adipocytes (32, 33). It has been reported that *PPARγ* is highly expressed in high-fat chickens and that its expression increases during preadipocyte differentiation (34, 35). It has also been demonstrated that *C/EBPβ* activates the expression of *C/EBPα* and *PPARγ* through synergy with *C/EBPδ* to complete the adipocyte differentiation process (30, 36, 37). The characteristics of fat deposition differ between birds and mammals, where the main site of lipid biosynthesis is the liver or adipose tissue, respectively (38–40). *APOA1* is a component of high-density lipoprotein (HDL), a molecule that transports cholesterol and phospholipids from other parts of the body through the bloodstream to the liver (41, 42). In this study, we demonstrated that *RGS16* overexpression in ICP-1 cells significantly increases the expression of *PPARγ*, *APOA1*, and *C/EBPβ* and promotes lipid droplet formation. At the same time, we found that the expression of *RGS16* in ICP-1 cells in-creases with an increasing duration of differentiation. These results demonstrate that *RGS16* may promote the differentiation of preadipocytes in influencing adipogenesis.

## 5. Conclusion

In summary, *RGS16* regulates abdominal lipid deposition by promoting ICP-1 cell differentiation and inhibiting ICP-1 cell proliferation. Eight *RGS16* SNPs were found to be significantly associated with fat-related traits, including AFW, AFR, and ST, in the Wens Sanhuang chicken population. Of the identified SNPs, rs73145592 and rs315021359 were situated within coding region, while the remaining six polymorphisms (rs735029742, rs738408023, rs735761612, rs741662332, rs16622145, and rs733023691) were located within the intronic region of RGS16. Furthermore, the haplotypes within the LD block were found to be significantly associated with AFR and ST. Finally, the findings our current study indicate that *RGS16* may play a significant role in the mechanism of abdominal fat accumulation in chickens. Therefore, it could be considered as a potential molecular marker to aid in the selection of broilers during breeding to improve their traits related to fat deposition.

## Data availability statement

The original contributions presented in the study are included in the article/Supplementary material, further inquiries can be directed to the corresponding author.

## Author contributions

MY and ZF: conceptualization. MY, QL, and SZ: methodology. MY: software, formal analysis, resources, data curation, writing—original draft preparation, and visualization. MY, KL, and YX: validation. ZF: investigation. LG and QL: writing—review and editing. QL: supervision, project administration, and funding acquisition. All authors have read and agreed to the published version of the manuscript..

## Funding

Funding for this project was provided by several sources, including the Key-Area Research and Development Program of Guangdong Province (2022B0202100002), the Guangdong Province Modern Agricultural Industry Technology System Project (2022KJ128, 2023KJ128), the Science and Technology Program of Chaozhou City (202101ZD07), and the National Key Research and Development Program of China (2022YFF1000201).

## Conflict of interest

The authors declare that the research was conducted in the absence of any commercial or financial relationships that could be construed as a potential conflict of interest.

## Publisher’s note

All claims expressed in this article are solely those of the authors and do not necessarily represent those of their affiliated organizations, or those of the publisher, the editors and the reviewers. Any product that may be evaluated in this article, or claim that may be made by its manufacturer, is not guaranteed or endorsed by the publisher.

## Footnotes

^1^ https://www.ncbi.nlm.nih.gov/tools/primer-blast/
