# Supplementary material for: Exploring the association between fat-related traits in chickens and the RGS16 gene: insights from polymorphism and functional validation analysis
Source: Front Vet Sci. 2023 May 10;10:1180797. doi: 10.3389/fvets.2023.1180797 (PMC10205986; doi:10.3389/fvets.2023.1180797)
Supplement: Supplementary file 1 [file Table_1.DOCX]

Table S1. Primers’ sequence information.

| **Primers’ name** | **Sequence (5’-3’)** | **Annealing temperature (℃)** |
| --- | --- | --- |
| *RGS16*-F | GAGGATCCGATGAGTTGCTGG | 60 |
| *RGS16*-R | TGAGTTCTTTGGCTCTCTCCA |  |
| *PPARγ*-F | TCCTTCCCGCTGACCAAA | 60 |
| *PPARγ*-R | TCCTGCACTGCCTCCACA |  |
| *APOA1*-F | GACAAGAACAGCAACGAGTACCGC | 60 |
| *APOA1*-R | CCTGAAGATGCCCCGCAGAGT |  |
| *C/EBPβ*-F | GCGGACTGTTTGGCTGCTCT | 60 |
| *C/EBPβ*-R | CGGGTGAGGCTGATGTAGGTGT |  |
| *CCNA1-*F | CACAACAGAAATGCGTGCCA | 60 |
| *CCNA1-*R | CAGGTAGTTCACCGCCAAGT |  |
| *CCND1-*F | CCTGCATGTTTGTGGCTTCC | 60 |
| *CCND1-*R | TTGCAGTAACTCGTCGGGTC |  |
| *CCNE1-*F | AAGAGGAAAGCCGATGTGGC | 60 |
| *CCNE1-*R | TCCAGGACAGCTGGTTTTCG |  |
| *GAPDH*-F | TCCTCCACCTTTGATGCG | 60 |
| *GAPDH*-R | GTGCCTGGCTCACTCCTT |  |
| S1-*RGS16*-F | CCACTGGGATGCCCCTTTACC | 58 |
| S1-*RGS16*-R | CATGTTTGGCAGGATGTCACC |  |
| S2-*RGS16*-F | CGTCCCAGCTCCACCAGTT | 58 |
| S2-*RGS16*-R | TCAACTGCCATGCTGAGATGC |  |
| S3-*RGS16*-F | CACCGCCTCCCAGTGCAAG | 58 |
| S3-*RGS16*-R | AGGACGCCCAGGACCTTGA |  |

Table S2 Association analysis of SNPs with fat-related traits in Wens Sanhuang chicken.

| **SNPs** | **Trait** | **MEAN ± SEM** | | | ***p*-value** |
| --- | --- | --- | --- | --- | --- |
| rs736356137 |  | CC (154) | TC (190) | TT (65) |  |
|  | AFW(g) | 96.69 ± 29.28 | 98.35 ± 30.16 | 97.09 ± 26.96 | 0.866 |
|  | AFR(%) | 7.63 ± 1.70 | 7.72 ± 1.83 | 7.62 ± 1.63 | 0.865 |
|  | ST (mm) | 7.62 ± 1.63 | 7.54 ± 1.77 | 7.14 ± 1.54 | 0.145 |
| rs740542560 |  | AA (284) | AG (109) | GG (16) |  |
|  | AFW(g) | 98.10 ± 29.27 | 95.54 ± 30.32 | 100.84 ± 22.31 | 0.665 |
|  | AFR(%) | 7.70 ± 1.71 | 7.55 ± 1.90 | 7.91 ± 1.19 | 0.614 |
|  | ST (mm) | 7.61 ± 1.68 | 7.25 ± 1.71 | 7.35 ± 1.48 | 0.155 |
| rs731768083 |  | CC (16) | GC (109) | GG (284) |  |
|  | AFW(g) | 100.84 ± 22.31 | 95.54 ± 30.32 | 98.1 ± 29.27 | 0.665 |
|  | AFR (%) | 7.91 ± 1.19 | 7.55 ± 1.90 | 7.70 ± 1.71 | 0.614 |
|  | ST (mm) | 7.35 ± 1.48 | 7.25 ± 1.71 | 7.61 ± 1.68 | 0.155 |
| rs737985212 |  | CC (291) | CT (111) | TT (7) |  |
|  | AFW (g) | 96.36 ± 29.03 | 99.97 ± 29.97 | 107.37 ± 28.79 | 0.364 |
|  | AFR (%) | 7.61 ± 1.73 | 7.79 ± 1.81 | 8.17 ± 1.46 | 0.483 |
|  | ST (mm) | 7.48 ± 1.56 | 7.56 ± 2.00 | 7.95 ± 1.44 | 0.714 |
| rs740327014 |  | AA (6) | GA (74) | GG (329) |  |
|  | AFW (g) | 105.70 ± 51.97 | 96.34 ± 25.54 | 97.65 ± 29.65 | 0.744 |
|  | AFR (%) | 8.10 ± 2.74 | 7.59 ± 1.59 | 7.68 ± 1.76 | 0.778 |
|  | ST (mm) | 6.95 ± 0.57 | 7.31 ± 1.44 | 7.56 ± 1.75 | 0.370 |
| rs741317696 |  | CC (273) | CT (129) | TT (7) |  |
|  | AFW (g) | 97.91 ± 29.20 | 97.13 ± 29.81 | 89.90 ± 25.46 | 0.762 |
|  | AFR (%) | 7.69 ± 1.72 | 7.64 ± 1.80 | 7.58 ± 1.75 | 0.955 |
|  | ST (mm) | 7.50 ± 1.63 | 7.50 ± 1.81 | 8.09 ± 1.80 | 0.655 |
| rs731255195 |  | AA (254) | GA (145) | GG (10) |  |
|  | AFW (g) | 96.85 ± 28.50 | 98.85 ± 30.57 | 95.44 ± 32.41 | 0.788 |
|  | AFR (%) | 7.65 ± 1.69 | 7.71 ± 1.83 | 7.59 ± 1.93 | 0.937 |
|  | ST (mm) | 7.57 ± 1.62 | 7.45 ± 1.81 | 6.71 ± 1.26 | 0.249 |
| rs738816885 |  | AA (257) | GA (142) | GG (10) |  |
|  | AFW (g) | 96.89 ± 28.56 | 98.83 ± 30.51 | 95.44 ± 32.41 | 0.798 |
|  | AFR (%) | 7.65 ± 1.69 | 7.71 ± 1.84 | 7.59 ± 1.93 | 0.927 |
|  | ST (mm) | 7.58 ± 1.63 | 7.44 ± 1.81 | 6.71 ± 1.26 | 0.230 |
| rs739467979 |  | CC (257) | TC (142) | TT (10) |  |
|  | AFW (g) | 96.89 ± 28.56 | 98.83 ± 30.51 | 95.44 ± 32.41 | 0.798 |
|  | AFR (%) | 7.65 ± 1.69 | 7.71 ± 1.84 | 7.59 ± 1.93 | 0.927 |
|  | ST (mm) | 7.58 ± 1.63 | 7.44 ± 1.81 | 6.71 ± 1.26 | 0.230 |
| rs731997801 |  | AA (255) | AG (143) | GG (11) |  |
|  | AFW (g) | 96.89 ± 28.65 | 98.9 ± 30.42 | 94.46 ± 30.91 | 0.757 |
|  | AFR (%) | 7.65 ± 1.70 | 7.72 ± 1.83 | 7.59 ± 1.83 | 0.921 |
|  | ST (mm) | 7.57 ± 1.62 | 7.45 ± 1.82 | 6.80 ± 1.23 | 0.301 |
| rs741182381 |  | CC (256) | TC (143) | TT (10) |  |
|  | AFW (g) | 96.79 ± 28.14 | 98.99 ± 31.19 | 95.44 ± 32.41 | 0.752 |
|  | AFR (%) | 7.64 ± 1.66 | 7.72 ± 1.89 | 7.59 ± 1.93 | 0.901 |
|  | ST (mm) | 7.59 ± 1.62 | 7.40 ± 1.81 | 6.71 ± 1.26 | 0.176 |
| rs735029742 |  | CC (322) | TC (83) | TT (5) |  |
|  | AFW (g) | 97.69 ± 29.24 | 95.97 ± 30.8 | 85.64 ± 4.58 | 0.606 |
|  | AFR (%) | 7.68 ± 1.70 | 7.60 ± 1.97 | 7.21 ± 0.50 | 0.779 |
|  | ST (mm) | 7.63 ± 1.60^A^ | 7.24 ± 1.68^A^ | 6.42 ± 0.85 | 0.043 |
| rs738408023 |  | CC (6) | TC (93) | TT (311) |  |
|  | AFW (g) | 86.03 ± 4.18 | 97.94 ± 32.20 | 97.18 ± 28.79 | 0.631 |
|  | AFR (%) | 7.22 ± 0.44 | 7.74 ± 2.04 | 7.64 ± 1.67 | 0.750 |
|  | ST (mm) | 7.00 ± 1.64 | 7.18 ± 1.64^A^ | 7.65 ± 1.60^A^ | 0.034 |
| rs735761612 |  | CC (166) | TC (196) | TT (48) |  |
|  | AFW (g) | 98.30 ± 31.12 | 97.98 ± 28.79 | 90.14 ± 24.88 | 0.208 |
|  | AFR (%) | 7.74 ± 1.83^A^ | 7.74 ± 1.70^B^ | 7.04 ± 1.53^AB^ | 0.034 |
|  | ST (mm) | 7.47 ± 1.67 | 7.53 ± 1.60 | 7.78 ± 1.50 | 0.512 |
| rs735218667 |  | AA (10) | GA (146) | GG (254) |  |
|  | AFW (g) | 105.97 ± 38.47 | 98.35 ± 30.01 | 96.18 ± 28.68 | 0.493 |
|  | AFR (%) | 8.22 ± 2.03 | 7.70 ± 1.71 | 7.61 ± 1.76 | 0.527 |
|  | ST (mm) | 8.24 ± 1.45 | 7.67 ± 1.66 | 7.43 ± 1.60 | 0.127 |
| rs317340874 |  | CC (14) | GC (143) | GG (253) |  |
|  | AFW (g) | 103.61 ± 36.16 | 98.30 ± 29.93 | 96.21 ± 28.73 | 0.563 |
|  | AFR (%) | 7.97 ± 1.90 | 7.71 ± 1.71 | 7.61 ± 1.76 | 0.695 |
|  | ST (mm) | 7.84 ± 1.49 | 7.71 ± 1.67 | 7.41 ± 1.59 | 0.165 |
| rs741662332 |  | CC (20) | TC (154) | TT (265) |  |
|  | AFW (g) | 83.10 ± 29.92^AB^ | 100.31 ± 29.77^A^ | 98.49 ± 29.2^B^ | 0.049 |
|  | AFR (%) | 6.80 ± 1.90^AB^ | 7.88 ± 1.75^A^ | 7.69 ± 1.74^B^ | 0.034 |
|  | ST (mm) | 6.84 ± 0.97 | 7.52 ± 1.57 | 7.58 ± 1.71 | 0.154 |
| rs731455923 |  | CC (249) | TC (162) | TT (28) |  |
|  | AFW (g) | 98.58 ± 28.83 | 98.53 ± 30.9 | 96.48 ± 29.13 | 0.937 |
|  | AFR (%) | 7.71 ± 1.70 | 7.74 ± 1.87 | 7.63 ± 1.72 | 0.949 |
|  | ST (mm) | 7.63 ± 1.61^A^ | 7.49 ± 1.65^B^ | 6.83 ± 1.76^AB^ | 0.047 |
| rs741115314 |  | CC (324) | CT (104) | TT (11) |  |
|  | AFW (g) | 97.93 ± 29.07 | 99.87 ± 30.61 | 99.38 ± 36.16 | 0.841 |
|  | AFR (%) | 7.68 ± 1.76 | 7.82 ± 1.73 | 7.77 ± 2.18 | 0.790 |
|  | ST (mm) | 7.53 ± 1.61 | 7.52 ± 1.68 | 7.44 ± 2.36 | 0.983 |
| rs734517162 |  | AA (11) | GA (146) | GG (282) |  |
|  | AFW (g) | 90.13 ± 28.62 | 98.50 ± 30.34 | 98.71 ± 29.25 | 0.640 |
|  | AFR (%) | 7.46 ± 1.93 | 7.76 ± 1.79 | 7.70 ± 1.75 | 0.839 |
|  | ST (mm) | 7.01 ± 1.00 | 7.42 ± 1.54 | 7.60 ± 1.71 | 0.321 |
| rs315021359 |  | CC (19) | TC (158) | TT (262) |  |
|  | AFW (g) | 83.25 ± 30.98^AB^ | 99.95 ± 29.66^A^ | 98.61 ± 29.21^B^ | 0.043 |
|  | AFR (%) | 6.84 ± 2.05^AB^ | 7.85 ± 1.74^A^ | 7.70 ± 1.74^B^ | 0.045 |
|  | ST (mm) | 6.96 ± 1.07 | 7.49 ± 1.59 | 7.59 ± 1.70 | 0.259 |
| rs16622145 |  | AA (287) | AG (140) | GG (12) |  |
|  | AFW (g) | 99.49 ± 30.16 | 97.54 ± 28.46 | 83.38 ± 25.03 | 0.165 |
|  | AFR (%) | 7.80 ± 1.80^A^ | 7.66 ± 1.65^B^ | 6.48 ± 1.71^AB^ | 0.036 |
|  | ST (mm) | 7.45 ± 1.73 | 7.70 ± 1.48 | 7.37 ± 1.28 | 0.331 |
| rs16622146 |  | CC (287) | CT (136) | TT (16) |  |
|  | AFW (g) | 99.49 ± 30.16 | 97.13 ± 28.31 | 90.39 ± 29.28 | 0.404 |
|  | AFR (%) | 7.80 ± 1.80 | 7.64 ± 1.63 | 6.99 ± 2.02 | 0.164 |
|  | ST (mm) | 7.45 ± 1.73 | 7.70 ± 1.50 | 7.42 ± 1.12 | 0.333 |
| rs16622147 |  | CC (107) | CT (214) | TT (118) |  |
|  | AFW (g) | 99.76 ± 31.40 | 99.43 ± 29.09 | 95.4 ± 28.73 | 0.429 |
|  | AFR (%) | 7.85 ± 1.85 | 7.73 ± 1.73 | 7.57 ± 1.74 | 0.479 |
|  | ST (mm) | 7.43 ± 1.78 | 7.60 ± 1.72 | 7.48 ± 1.34 | 0.612 |
| rs16622148 |  | CC (15) | TC (136) | TT (288) |  |
|  | AFW (g) | 93.85 ± 26.70 | 97.13 ± 28.31 | 99.28 ± 30.32 | 0.651 |
|  | AFR (%) | 7.21 ± 1.87 | 7.64 ± 1.63 | 7.78 ± 1.82 | 0.385 |
|  | ST (mm) | 7.50 ± 1.10 | 7.70 ± 1.50 | 7.45 ± 1.72 | 0.331 |
| rs733023691 |  | CC (22) | TC (178) | TT (239) |  |
|  | AFW (g) | 114.96 ± 33.61^AB^ | 96.65 ± 29.60^B^ | 98.23 ± 28.80^A^ | 0.023 |
|  | AFR (%) | 8.60 ± 1.90^AB^ | 7.60 ± 1.78^B^ | 7.72 ± 1.72^A^ | 0.042 |
|  | ST (mm) | 7.50 ± 1.44 | 7.56 ± 1.59 | 7.50 ± 1.70 | 0.935 |
| rs312359940 |  | AA (45) | AT (188) | TT (206) |  |
|  | AFW (g) | 100.18 ± 29.63 | 98.35 ± 29.32 | 98.12 ± 29.90 | 0.914 |
|  | AFR (%) | 7.88 ± 1.73 | 7.71 ± 1.74 | 7.69 ± 1.79 | 0.797 |
|  | ST (mm) | 7.17 ± 1.86 | 7.51 ± 1.74 | 7.62 ± 1.49 | 0.245 |
| rs734891921 |  | CC (173) | TC (223) | TT (43) |  |
|  | AFW (g) | 98.31 ± 28.41 | 98.36 ± 29.63 | 99.28 ± 34.18 | 0.980 |
|  | AFR (%) | 7.67 ± 1.69 | 7.74 ± 1.77 | 7.78 ± 2.05 | 0.887 |
|  | ST (mm) | 7.51 ± 1.70 | 7.54 ± 1.65 | 7.52 ± 1.34 | 0.978 |
| rs80763227 |  | CC (126) | CT (212) | TT (101) |  |
|  | AFW (g) | 100.41 ± 30.75 | 99.06 ± 30.07 | 94.62 ± 26.80 | 0.311 |
|  | AFR (%) | 7.89 ± 1.83 | 7.70 ± 1.78 | 7.54 ± 1.62 | 0.337 |
|  | ST (mm) | 7.42 ± 1.76 | 7.60 ± 1.71 | 7.50 ± 1.33 | 0.610 |
| rs15902064 |  | CC (370) | CT (66) | TT (3) |  |
|  | AFW (g) | 98.82 ± 29.38 | 96.28 ± 31.37 | 97.77 ± 3.42 | 0.814 |
|  | AFR (%) | 7.69 ± 1.73 | 7.85 ± 1.98 | 7.81 ± 0.97 | 0.795 |
|  | ST (mm) | 7.55 ± 1.63 | 7.39 ± 1.75 | 7.23 ± 1.19 | 0.742 |

Table S3. Association analysis of block with fat-related traits in Wens Sanhuang chicken.

| **LD block** | **SNPs** | **Haplotype** | **Diplotype (n)** | **Fat-related traits** | | |
| --- | --- | --- | --- | --- | --- | --- |
|  |  |  |  | **AFW (g)** | **AFR (%)** | **ST (mm)** |
| Block 1 | rs735029742  rs738408023  rs735761612 | H1:CTC  (0.515)  H2:CTT  (0.355)  H3:TCC  (0.111) | H1H1 (104)  H1H3 (48)  H2H1 (158)  H2H2 (47)  H2H3 (33)  H3H3 (4) | 99.30 ± 31.26  94.56 ± 30.23  97.98 ± 28.12  90.14 ± 24.88  98.45 ± 32.80  84.63 ± 4.60 | 7.74 ± 1.78^B^  7.61 ± 1.94  7.77 ± 1.61^A^  7.04 ± 1.53^AB^  7.58 ± 2.10  7.11 ± 0.52 | 7.63 ± 1.66  7.30 ± 1.72  7.63 ± 1.60  7.78 ± 1.50^AB^  7.04 ± 1.60^B^  6.05 ± 0.23^A^ |
| *P*-value | | | | 0.4677 | 0.2004 | 0.0957 |
| Block 2 | rs741662332  rs731455923  rs315021359  rs16622145 | H1:TCTA  (0.339)  H2:TTTA  (0.247)  H3:CCCA  (0.216)  H4:TCTG  (0.186) | H1H1 (52)  H1H2 (76)  H1H3 (59)  H1H4 (56)  H2H2 (28)  H2H3 (48)  H3H3 (17)  H4H2 (37)  H4H3 (44)  H4H4 (12) | 100.66 ± 27.12^D^  100.84 ± 32.13^B^  105.41 ± 29.16^A^  100.84 ± 27.75^C^  96.48 ± 29.13  98.08 ± 30.62^E^  80.84 ± 29.41^ABCDE^  95.14 ± 29.15  96.47 ± 29.06  83.38 ± 25.03^A^ | 7.85 ± 1.59^D^  7.98 ± 1.89^B^  8.04 ± 1.66^A^  7.74 ± 1.65^E^  7.63 ± 1.72  7.68 ± 1.94^F^  6.66 ± 1.90^ABCDEF^  7.35 ± 1.69  7.87 ± 1.63C  6.48 ± 1.71^ABCDEF^ | 7.91 ± 1.89^B^  7.35 ± 1.72  7.59 ± 1.68^D^  7.94 ± 1.59^A^  6.83 ± 1.76^ABCD^  7.50 ± 1.68  6.80 ± 0.95^ABC^  7.77 ± 1.46^C^  7.40 ± 1.34  7.37 ± 1.28 |
| *P*-value | | | | 0.1081 | 0.0317 | 0.0496 |
